# Tools for loading MEDLINE into a local relational database

**DOI:** 10.1186/1471-2105-5-146

**Published:** 2004-10-07

**Authors:** Diane E Oliver, Gaurav Bhalotia, Ariel S Schwartz, Russ B Altman, Marti A Hearst

**Affiliations:** 1 Department of Genetics, Stanford University, Stanford, CA, USA; 2 Computer Science Division, University of California, Berkeley, CA, USA; 3 School of Information Management & Systems, University of California, Berkeley, CA, USA

## Abstract

**Background:**

Researchers who use MEDLINE for text mining, information extraction, or natural language processing may benefit from having a copy of MEDLINE that they can manage locally. The National Library of Medicine (NLM) distributes MEDLINE in eXtensible Markup Language (XML)-formatted text files, but it is difficult to query MEDLINE in that format. We have developed software tools to parse the MEDLINE data files and load their contents into a relational database. Although the task is conceptually straightforward, the size and scope of MEDLINE make the task nontrivial. Given the increasing importance of text analysis in biology and medicine, we believe a local installation of MEDLINE will provide helpful computing infrastructure for researchers.

**Results:**

We developed three software packages that parse and load MEDLINE, and ran each package to install separate instances of the MEDLINE database. For each installation, we collected data on loading time and disk-space utilization to provide examples of the process in different settings. Settings differed in terms of commercial database-management system (IBM DB2 or Oracle 9i), processor (Intel or Sun), programming language of installation software (Java or Perl), and methods employed in different versions of the software. The loading times for the three installations were 76 hours, 196 hours, and 132 hours, and disk-space utilization was 46.3 GB, 37.7 GB, and 31.6 GB, respectively. Loading times varied due to a variety of differences among the systems. Loading time also depended on whether data were written to intermediate files or not, and on whether input files were processed in sequence or in parallel. Disk-space utilization depended on the number of MEDLINE files processed, amount of indexing, and whether abstracts were stored as character large objects or truncated.

**Conclusions:**

Relational database (RDBMS) technology supports indexing and querying of very large datasets, and can accommodate a locally stored version of MEDLINE. RDBMS systems support a wide range of queries and facilitate certain tasks that are not directly supported by the application programming interface to PubMed. Because there is variation in hardware, software, and network infrastructures across sites, we cannot predict the exact time required for a user to load MEDLINE, but our results suggest that performance of the software is reasonable. Our database schemas and conversion software are publicly available at .

## Background

MEDLINE is a large biomedical bibliographic database that is well known to users around the globe. It contains over 12 million citations from over 4,600 journals. MEDLINE is a rich source of biomedical text that lends itself well to research on text mining, information extraction, and natural language processing in biomedical domains. The usual way in which users query MEDLINE is through PubMed, the web-based interface and search engine provided by the National Library of Medicine (NLM) [1]. PubMed allows individuals to conduct searches directly by entering search terms on web pages and viewing results, and supports software-based queries across the Internet with programming utilities offered by the NLM [2]. Because we were interested in developing custom-made programs that query MEDLINE, the programming utilities offered by the NLM were an obvious choice to consider. However, due to risks of server overload, the NLM places limits on the number of queries that a user can send in a given time interval, and requests that large-volume queries be done on nights or weekends [3]. By contrast, a local version of MEDLINE gives software developers greater control over how they use the data, and facilitates the development of customizable interfaces. In this report, we describe the design and implementation of the database schema and database loading tools we have built to enable others to produce similar systems at their sites.

The entire content of MEDLINE is available as a set of text files formatted in XML (eXtensible Markup Language) [4]. The NLM distributes these files at no cost to the licensee, but the files are large and not easily searched without additional indexing and search tools. For example, in the 2003 release of MEDLINE, there are 396 files (which cover citations through 2002), and the total uncompressed size of these files is 40.8 gigabytes (GB). Although it is relatively inexpensive to store 40.8 GB of data, it is not easy to manipulate data of that magnitude without good software support. Relational databases are a natural choice for storing MEDLINE because they are able to handle large amounts of data, offer built-in approaches to query optimization, and enable the developer to create indexes. Additionally, the standard query language for relational databases, SQL (Structured Query Language), enjoys widespread familiarity and can be integrated with text-database queries in some commercial systems.

Alternatives to relational databases are XML-based databases, which have recently emerged as another option for storing information transmitted in XML format.XML databases may exist as standalone databases or as add-ons to relational systems. The MEDLINE data set would be an excellent test of the capabilities of these databases because of its size and complexity. We focused on relational databases because they are currently more ubiquitous and standardized, and interested users are more likely to be comfortable with relational database technology.

In the remainder of this report, we describe the software tools we developed for converting MEDLINE in XML files to MEDLINE in a relational database, and provide a few sample queries that demonstrate the flexibility of the resulting system.

## Implementation

### Database schema

The NLM provides a DTD (Document Type Definition) that defines the structure of data in the MEDLINE XML files [5-7]. From this DTD, we designed a relational database schema. Although developers of MEDLINE at the NLM maintain their own version of MEDLINE in a relational database, the schema they use is not directly applicable to our purposes, because their implementation contains tables and data that are used for maintenance and that are not relevant to external users. Thus, it was appropriate for us to design our own schema based on the specific content of the XML files, as defined by the DTD. Other groups currently license the same MEDLINE XML files and may have implemented all of MEDLINE in a relational database, but if so, their database schemas are not well publicized and were not available.

There are multiple ways in which one can design a schema from the same DTD, because DTD elements and attributes can be mapped to tables and fields in different ways. Certain design decisions may favor speed at loading time, and others may favor speed and ease of use at query time. Loading records associated with 12 million citations into a database is very time consuming, and the time can be minimized if lookups to the database are minimized during loading. In general, we aimed to minimize lookups even if that meant repeating information in the database. As developers often do in the design of data warehouses, we chose to de-normalize the schema in order to improve read-only query performance, which is the typical data access pattern in our workload.

**Figure 1:**
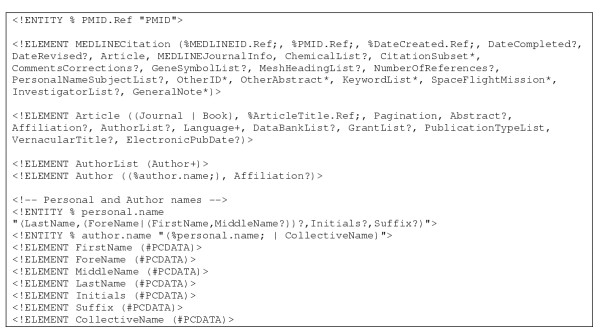
**Representation of information related to authors in the DTD. **Selected portions of the DTD are shown. Database schema designers determine how entities and elements are converted to table names or field names in the database schema. See Figure 2 for the author table.

**Figure 2:**
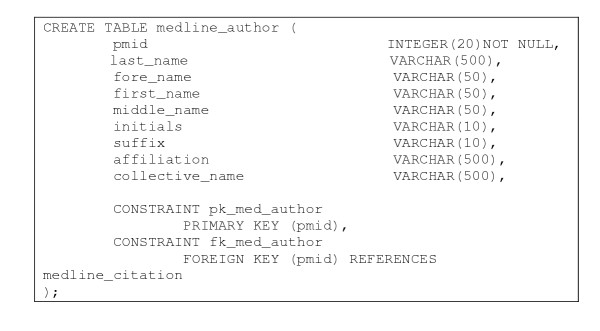
**Representation of author information in the database schema. **The typical table has a PubMed identifier (pmid) associated with other fields.

The typical table contains a PubMed identifier (PMID) in one column, and data related to that PMID in the remaining columns. Figures 1 and 2 show original representation of content from a portion of the DTD and a corresponding table that follows the typical table structure.

Our development team included a group of researchers from the University of California at Berkeley and another group from Stanford University. We shared similar goals in that we all wanted to load MEDLINE into a relational database, but because we were in two different departments at two different institutions, we had different project constraints and timelines. Thus, our groups were loosely associated in the software development process, but not closely integrated, and therefore, the original schema that we shared diverged.

The result was three MEDLINE schemas and three software variants: One schema was used with Java code developed at Berkeley, another schema was used with Berkeley's code modified to run at Stanford, and the third schema was used with Perl code developed at Stanford. Here we describe the underlying design that influenced all three of the schemas. (The schema used for the Java program did not include information from DTD elements DataBankList and AccessionNumberList. This has been corrected in the most recent version of the software available on the Berkeley website.)

The main table in the schema is *medline_citation*. The *medline_citation *table contains the PMID as the primary key and has additional columns that correspond to single-valued elements in the DTD, where the values of those elements depend on the PMID. The *medline_abstract *table is similar in that it has a PMID as the primary key and columns of data that depend on the PMID. Since document abstracts are larger than the other data types, we placed them in a separate table. However, since abstracts are stored as CLOBs (Character Large Objects), they are not stored in the same pages as the rest of the fields in the *medline_abstract *table. Therefore, in a more recent implementation, we removed the *medline_abstract *table from the schema, and added the *abstract_text *field as a CLOB in the *medline_citation *table. This change reduces the number of tables by one, and eliminates the need for a join between the *medline_citation *and *medline_abstract *tables.

Some tables in the schema have more than one row corresponding to the same PMID. Columns in these tables map to multi-valued elements in the DTD. Examples are the table *medline_keyword_list*, which stores multiple values of *keyword *for a given PMID, and *medline_gene_symbol_list*, which stores multiple values of *gene_symbol *for a given PMID.

The element *Article *in the DTD has a one-to-one relationship between an article and a PubMed identifier. Rather than giving *Article *its own table, we put single-valued data from *Article *into the table *medline_citation*.

To keep track of the name of the file from which data are read for a given citation, we added the field *xml_file_name *to the *medline_citation *table. This field does not correspond to any element in the DTD structure, but allows the database administrator to go back to the original XML file if necessary to find the original source of the data.

We could have stored each author only once in a table of its own, and assigned each author a unique integer primary key to serve as an author identifier. An author is represented by a combination of values in fields for last name, forename, first name, middle name, initials, suffix, affiliation, and collective name. Another table would have stored the set of author identifiers associated with each PMID, and because integer joins are fast, this design would have facilitated rapid search for all PMIDs associated with a given author, by joining the author table with the table of author identifiers and citations. However, there are several drawbacks to this approach. Generating integer primary keys during loading requires that either a lookup be done to see if each author of each citation already exists or not (35 million lookups), or all authors and primary keys must be kept in memory. The former approach is very time consuming during loading; the latter approach strains memory resources. In addition, regardless of how primary keys are managed during loading, it is not possible to determine algorithmically if two different representations of one author are actually the same author, or if one representation is actually two different authors. We therefore avoided generating unique primary keys and repeated all eight fields representing the author for every citation occurrence of that author.

**Figure 3:**
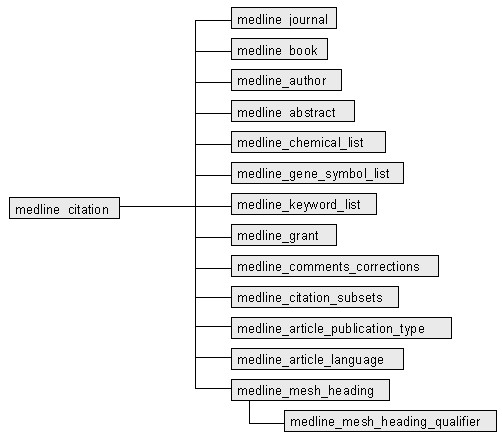
**Dependencies in the database schema. **Parent tables contain primary keys that child tables reference as foreign keys. The main table *medline_citation*, is a parent of thirteen other tables. The table *medline_mesh_heading *is a parent of *medline_mesh_heading_qualifier*.

Figure 3 shows relationships among the tables. The table *medline_journal *is a parent of thirteen other tables (it contains the primary key *pmid*, which is used as a foreign key by the other tables). One of the other tables, *medline_mesh_heading*, is a parent of *medline_mesh_heading_qualifier*. Multiple qualifiers can be associated with each MeSH heading for a given citation.

### Parsing and loading software

We implemented three versions of software that parses and loads MEDLINE. The first was Java MedlineParser, which was developed at Berkeley [see additional file 1]. The second was the same Java code, modified to run at Stanford. The third was Perl ParseMedline, which was developed at Stanford.

**Figure 4:**
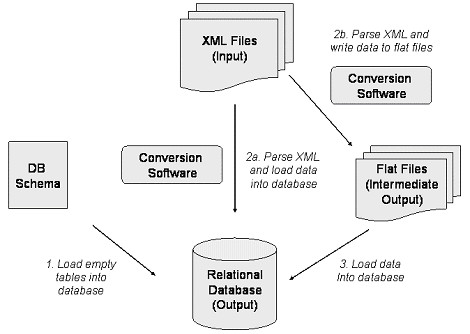
**MEDLINE database development process. **In Step 1, the user loads the schema, creating empty tables in the database. In Step 2, the conversion software parses the XML files and either loads the data directly into the database (2a), or writes the data out to intermediate text files (2b). If intermediate text files are generated, data from those files are loaded into the database as a separate step in Step 3.

All versions of the software perform two basic tasks: (1) they parse the XML files to collect data, and (2) they load the data into the database. Figure 4 shows the steps involved. Data can be loaded as they are collected, or can be written out to disk initially, and loaded later. All three versions offer these two options to the user. Document parsing is processor intensive, data insertion is disk intensive, and if needed, the two tasks can be executed at different times to accommodate other demands on the server.

There are two types of application programming interfaces (APIs) for parsing XML files – the tree-based DOM (Document Object Model) and the event-based SAX (Simple API to XML) [8]. We chose the latter. A DOM parser organizes data from the XML file into a tree of nodes, and requires that the entire document be read in and stored in memory prior to writing out any data. Thus, the DOM parser is impractical for large documents whose data do not fit in memory. The SAX parser, however, receives data through a stream, and recognizes the beginning or end of a document, element, or attribute in an event-driven manner. It writes out data as it proceeds through the parsing process, and there is no need for the entire document to fit into memory. In XML MEDLINE, one document is a single XML-formatted MEDLINE file, and in the 2003 release, the majority of files range in size from 60 to 142 megabytes (MB). Using the DOM parser would put great demand on resources. In addition, the SAX parser is faster because it does not need to create an entire XML tree structure, map that structure to the program's data structures, and then throw out the original tree. Instead, it creates its own data structures as events are handled.

The Java version uses the Java SAX parser to parse the XML files, and JDBC (Java Database Connectivity) to communicate with the database. The Perl version uses the Perl SAX parser, and Perl DBI (Database Interface) to communicate with the database. We provide additional detail about the Java implementation here.

The SAX parser requires the developer to write code that specifies the data model for objects in the domain. The data model is an object model that represents tables in the schema. The SAX parser also requires code that listens for SAX events and that maps elements – or nodes in the XML tree – to the object model. We created two main classes upon which our code is based: GenericXMLParser and NodeHandler. GenericXMLParser is responsible for generating events when nodes that correspond to objects in the object model are encountered in the document, and NodeHandler provides the event listener. Together, these two classes form a generic approach to reading in XML data and writing out those data to tables; they are independent of the DTD or table structure.

As the parser processes the document, it decides how to handle the semantics of data at each node and determines whether to store parsed data at that node or to delegate the event to a child handler. For each node that corresponds to a table, there is a handler class that extends NodeHandler. A handler defines metadata for the node, and encodes any non-standard behavior at that node. An example of metadata is shown in Table 1. Metadata include column names for the table and an XML element associated with each column name. An XML element is represented by a concatenation of the name of the element that holds the data value, and elements higher up in the element stack up to the node that corresponds to the object, or table. This concatenated name gives a unique representation of the element that holds the data. Finally, the data type is given for each column. The column names and data types match those specified in the database schema.

Since NodeHandler and GenericXMLParser are generic, they can be used to write similar parsers for other XML documents. We have, for example, used these classes to write a parser for MeSH (Medical Subject Headings) XML files, which are also distributed by the NLM. The MeSH files are small compared to MEDLINE. MeSH 2003 comes in three XML files that total less than 600 MB.

An optional feature is validation. XML files provided by the NLM are validly formatted, but we provide additional checks to ensure that all element tags in the XML data file have been handled by the parser and that all data have been inserted into the database. A developer who is extending the software to cover new tables can use this feature to ensure that metadata definitions are correct in classes that extend NodeHandler.

### Choice of Relational Database Management Systems

In the course of our work, we applied the software tools we were developing to three different relational database products. Our Berkeley team initially experimented with PostgreSQL, since PostgreSQL is an open-source relational database and is freely available and modifiable. For the final implementation, however, we chose IBM's DB2 8.1 over PostgreSQL because we found that it could load our data more efficiently and because DB2 has a text-search extender. Our Stanford team used Oracle 9i, which like DB2, offers word-based indexing of text fields. Word-based indexing is essential to support keyword search of MEDLINE titles and abstracts.

### Hardware configurations

At Berkeley, we used a Pentium IV Intel Xeon 2.00-GHz dual-processor system, with 1 GB of random access memory (RAM). It had an Integrated Drive Electronics (IDE) hard disk with a rotational speed of 7200 revolutions per minute (RPM). At Stanford, we used a Sun Fire V880 server configured with four 750-MHz processors, 8 GB of RAM, and storage-area-network (SAN) storage for the relational database. We also used a Sun Enterprise 3500 server with eight 400-MHz processors and 4 GB of RAM for reading input files and writing intermediate output files in the Perl version.

## Results and discussion

In this section, we describe loading time and disk-space utilization for the three implementations, followed by examples of queries, emphasizing differences between our system and PubMed.

The first implementation used the Java software, run on an Intel system (Linux), using IBM's DB2 database management system. The second implementation also used the Java software, run on a Sun server (SunOS), using Oracle's 9i database-management system. The third implementation used the Perl program, run on networked Sun servers, also using Oracle 9i. Table 2 summarizes our results.

### Loading time and disk space utilization

It took 76 hours (3 days and 4 hours) for the Berkeley group to run Java MedlineParser to load MEDLINE, and 196 hours (8 days and 4 hours) for the Stanford group to do so in Oracle. There were numerous differences between the two systems, and it was not possible to test each variable independently. Therefore, we present our data as a range of possibilities, and recognize that other users will have systems that are not the same as either of ours. We believe that differences in processor speed, memory, disk read-write efficiency, and optimization methods employed in commercial database-management systems may have affected loading times. In addition, the code diverged slightly after the initial transfer of code from Berkeley to Stanford, with the main difference being that the Berkeley version used CLOBs for abstracts, whereas the Stanford version used text fields truncated to 4000 characters (size limit imposed on VARCHAR datatype). The Stanford run was also slower because a log file was generated, whereas this feature was turned off in the Berkeley run.

The space requirement for the DB2 instance of MEDLINE at Berkeley was 46.3 GB, of which 10.4 GB are consumed by the abstract text CLOBs, 18.1 GB by the other tables, and 17.8 GB by indexes. The space requirement for the Oracle instance of MEDLINE at Stanford was 37.7 GB. The difference in size is primarily due to differences in the number of records that were loaded. The Berkeley group loaded data from XML files that included all of 2002 (early 2003 release) but also included additional files through April 2003. The Stanford group loaded data from 2002 XML files only. Berkeley parsed and loaded 500 input files (44.4 GB); Stanford parsed and loaded 396 input files (40.8 GB).

The Stanford group used Perl ParseMedline to load an additional instance of MEDLINE. Parsing and loading of this instance of the database took place in a two-stage process. In the first stage, Perl ParseMedline parsed the XML files and wrote the data to disk in comma-separated-value files. To reduce processing time, the 396 XML input files were divided into 8 sets of about 50 files each, and sets were processed in parallel. The maximum time required for processing one set was 31 hours (1 day and 7 hours). The output comma-separated-value files required 25.6 GB of disk space. In the second stage, the Stanford team loaded data from the comma-separated-value files into the Oracle database using SQL*Loader, a data loading tool provided by Oracle. This stage took 33 hours (1 day and 9 hours) and used 31.6 GB of space in the Oracle database. This version used less space than the other two primarily because it had less indexing and fewer key constraints. Relaxation of constraints is reasonable because the data are well curated by the NLM, and we can count on data in the XML files released by the NLM to be of high quality.

The total time required to parse and load the files in this two-stage process is the sum of the time required to parse the largest file if all files are processed in parallel (first stage) and the time required to load the resulting comma-separated-value files into the database (second stage). Alternatively, if the input files are parsed in series, the time for the first stage would be the sum of the input-file processing times. In our case, we overlapped the runs in a way that was convenient for us, given space and user constraints, and therefore mixed the parallel and serial approach. The overall time for our first stage was 99 hours (4 days and 3 hours); adding this time to the second stage gave a total time of 132 hours (5 days and 12 hours). Given the length of time to process each of our eight batches, we can estimate a lower limit of 64 hours (2 days and 16 hours), and an upper limit of 253 hours (10 days and 13 hours), if we had run the files completely in parallel or in series, respectively.

### Sample queries

Certain queries that cannot be done easily through the PubMed application programming interface (API) can be done in a single SQL query to our relational database. In this section, we show the results of a several sample queries, run on a version of MEDLINE that contains citations through April 2003.

Timing data is presented in terms of "cold" caches and "warm" cache. The cold cache represents the worst case for timing, assuming the database server has just been restarted and the buffer pool is empty. The warm cache represents the best possible performance: running the same query a second time. A typical timing number should fall somewhere between the two; hence these times represent the range of expected times to run the sample queries.

A very simple query is one that retrieves all PMIDs in MEDLINE, where *pmid *is a column in table *medline_citation *(Table 3).

Although typical users of PubMed would not be interested in such a query, we are managing MEDLINE as a complete database, and need to have access to all PMIDs. Running this query on the Berkeley implementation took 12 minutes and 26 seconds.

Many articles in MEDLINE are assigned terms from MeSH. Another capability of this system that distinguishes it from PubMed is the ability to rank order journals according to how many articles those journals have published that have been assigned a particular MeSH term. In the query shown below (Table 4), the number of publications indexed with the MeSH term (or *descriptor_name*) "Leukemia" is shown for each journal (where *medline_ta *is the title abbreviation of a journal).

The result of this query is a table consisting of journals paired with number of publications (Table 5); note that the query does not normalize for the fact that some journals have been publishing for more years than others, and publish more articles than others. This query ran in 4 minutes with a cold cache, and in 20 seconds with a warm cache.

SQL includes the "LIKE" operator which allows for partial matches. By modifying the query above to change the fifth line to read "WHERE msh.descriptor_name LIKE 'Leukemia%'," we change the query to match all MeSH terms that *begin *with "Leukemia". The query would thus include terms such as "Leukemia, Subleukemic" and "Leukemia, Feline." This results in dramatically more results, although the rank ordering is not all that different (Table 6). This query ran in 4 minutes with a cold cache, and in 46 seconds with a warm cache.

MeSH terms are organized into a hierarchy, and each MeSH term has associated with it one or more descriptor tree numbers that indicate its place in the hierarchy. The Berkeley group developed additional code to parse MeSH XML data files (which can be downloaded from the NLM website [9]), and added MeSH tree data to the MEDLINE database. Using the additional functionality provided by the MeSH hierarchy, we can modify the query above to rank order journals according to how often they have articles that have been assigned the MeSH term under a certain tree number, thus eliminating the sensitivity to different spellings of related concepts that was shown in the queries above. In MeSH, a child tree number shares its leftmost digits with its parent tree number, and differs in its three rightmost digits Therefore, the SQL "LIKE" operator can be used to find a MeSH term and its descendants, as shown in the query below (Table 7). The MeSH tree number for "Leukemia" is "C04.557.337".

The results of this query are shown in Table 8.

This query ran in 4 minutes with a cold cache, and in 41 seconds with a warm cache.

The DB2 version of the system implementation makes use of the text index that is incorporated into the RDBMS system, using the operator "CONTAINS" which is not part of standard SQL. The following query asks how many papers in the last three years of MEDLINE have been published by authors with affiliations at Berkeley or Stanford (Table 9).

This yields the results in Table 10.

This query ran in 2.5 minutes with a cold cache, and in 7 seconds with a warm cache.

When we ran a similar query to determine the number of articles published by Berkeley, Stanford, MIT, Yale, and Harvard, the increase in time to run the query was minimal. This modified query ran in 3 minutes and 35 seconds with cold cache, and in 15 seconds with warm cache.

Thus, SQL makes it easy to quantify and rank order results, and does not require a post-processing step as would be necessary with queries to the PubMed API. Similarly, results retrieved from previous queries can be stored directly in the same database, and reused in later queries by simply joining MEDLINE tables with user-created tables. Again, the power of SQL may alleviate the need for a post-processing step. Instead of writing custom code to integrate results from the current query to PubMed with results from previous queries, the user could use SQL joins to integrate current and previous results.

Although our system offers capabilities that the PubMed API does not, we point out that PubMed offers functionality that is not available in our system. For example, the "Related Articles" feature found in PubMed is not available, and links to full text are not available. Also, PubMed provides a user interface that is more intuitive than SQL for an end user who is not a database expert, and will be preferred by users who simply want to look up an article.

The value of our system is that it offers greater flexibility for innovative software developers who want to experiment with novel techniques for searching biomedical text, and for system developers who want to build systems of which MEDLINE is a component. If such developers want to offer their systems to end users (e.g., biologists, clinicians, or the lay public), they will need to create more intuitive user interfaces. With direct access to the underlying database, developers can create interfaces that are specifically designed to serve the needs of their particular users.

## Conclusions

In this work, we developed highly customizable Java parsing code and a relational database schema that others may be interested in using or modifying. We developed software that uses the Java programming language and the SAX parser to parse XML-formatted MEDLINE files and load the data into a relational database. We loaded one copy into DB2 and another into Oracle, using our Java tool.

We also created a similar tool in Perl. The Perl code is less flexible and not as readily extensible as the object-oriented code of our Java software, but the functionality offered by the resulting database implementations is very similar.

Differences in loading time among the three installations of MEDLINE were due to a multiple factors, including differences in processors, disk storage devices, memory, operating systems, database-management systems, methods implemented in the software, and choices made by the user. Factors that affected disk-space utilization included the fact that one group loaded more data than the other, abstracts were stored as CLOBs at one site and as truncated text at the other, and indexes differed. Other groups will have system setups that differ from ours, and may make their own modifications to the code that affect their loading times. By presenting data on three examples, we have demonstrated a range of performance results as a guide to what other users might expect at their sites.

Future work includes adding functionality to update the system to new versions of MEDLINE, and to accommodate MEDLINE update files. The Stanford group has begun to use MEDLINE to extract drug-gene relationships from the literature, and the Berkeley group used the system, augmented with data from MeSH and LocusLink, to compete in the TREC 2003 genomics track competition [10]. As we continue to use these systems for research purposes, we are likely to identify alternative approaches that offer enhancements and improvements over the current design. We encourage others who work in similar areas to contribute to the open-source effort.

An updated version of our Java code accepts MEDLINE XML input files released in early 2004 that conform to the latest DTD (November 2003). The open-source code for this most current version of MedlineParser is available at .

## Availability and requirements

Project name: Java MedlineParser

Project web page: 

Operating system: Platform independent

Programming language: Java

Other requirements: Java 1.4.1 or higher, JAXP, relational database, and JDBC driver appropriate for the particular target database

License: None

Any restrictions on use by non-academics: None

Project name: Perl ParseMEDLINE

Project web page: 

Operating system: Platform independent

Programming language: Perl

Other requirements: Perl 5.8 or higher to handle MEDLINE Unicode data (if writing directly to database), or earlier version of Perl (if writing to comma-separated-value files first), Perl modules DBI and XML::Parser::PerlSAX, relational database, and Perl database driver appropriate for the particular database (e.g., DBD::Oracle)

License: None

Any restrictions on use by non-academics: None

## Authors' contributions

DO, GB, and AS, developed the MEDLINE database schemas. GB and AS designed and implemented the Java MedlineParser. GB and AS ran MedlineParser to install MEDLINE in DB2 at Berkeley. DO ran MedlineParser to install MEDLINE in Oracle 9i at Stanford. DO developed Perl ParseMedline and ran it to install the second version of MEDLINE at Stanford. DO and GB were primary authors of the article, and the remaining authors added their contributions to the manuscript. MH supervised the work at Berkeley; RB supervised the work at Stanford.

## Figures and Tables

**Table 1:** Metadata for medline_author table.

**columnNameDef**	**xmlElementNameDef**	**columnTypeDef**
pmid	PMID	Types.INTEGER
last_name	Author.LastName	Types.VARCHAR
fore_name	Author.ForeName	Types.VARCHAR
first_name	Author.FirstName	Types.VARCHAR
middle_name	Author.MiddleName	Types.VARCHAR
initials	Author.Initials	Types.VARCHAR
suffix	Author.Suffix	Types.VARCHAR
affiliation	Author.Affiliation	Types.VARCHAR
collective_name	Author.CollectiveName	Types.VARCHAR

***columnNameDef***: column names for the table,

***xmlElementNameDef***: XML element names that correspond to column names,

***columnTypeDef***: data type of each column

**Table 2:** Loading time and disk-space utilization.

Site	Language	Processor	Database	Input Size	Loading Time	Disk Space
Berkeley	Java	Intel	DB2	44.4 GB (500 files)	76 hours	46.3 GB
Stanford	Java	Sun	Oracle	40.8 GB (396 files)	196 hours	37.7 GB
Stanford	Perl	Sun	Oracle	40.8 GB (396 files)	132 hours	31.6 GB

**Table 3:** 

SELECT pmid
FROM medline_citation

**Table 4:** 

SELECT	mc.medline_ta, count(mc.pmid) as num_of_publications
FROM	medline_citation mc
	JOIN medline_mesh_heading msh ON
	mc.pmid = msh.pmid
WHERE	msh.descriptor_name = 'Leukemia'
GROUP BY	mc.medline_ta
ORDER BY	count(mc.pmid) desc
FETCH	first 10 rows only;

**Table 5:** 

Blood	940
Cancer	619
Rinsho Ketsueki	610
Cancer Res	588
Br J Haematol	524
Bone Marrow Transplant	520
Lancet	515
Leuk Res	476
Leukemia	463
The N Engl J Med	342

**Table 6:** 

Blood	6721
Cancer Res	4653
Leukemia	4640
Br J Haematol	3918
Leuk Res	3061
Cancer	2772
Rinsho Ketsueki	2628
Cancer Genet Cytogenet	2192
Bone Marrow Transplant	2123
Lancet	1931

**Table 7:** 

SELECT	mc.medline_ta, count(mc.pmid) as num_of_publications
FROM	medline_citation mc
	JOIN medline_mesh_heading msh ON
	mc.pmid = msh.pmid
	JOIN mesh_descriptor md ON
	md.descriptor_name = msh.descriptor_name
	JOIN mesh_desc_tree_number mdtn ON
	md.descriptor_ui = mdtn.descriptor_ui
WHERE	mdtn.tree_number LIKE 'C04.557.337%'
GROUP BY	mc.medline_ta
ORDER BY	count(mc.pmid) desc
FETCH	first 10 rows only;

**Table 8:** 

Blood	7361
Leukemia	5168
Cancer Res	4595
Br J Haematol	4249
Leuk Res	3274
Cancer	2856
Rinsho Ketsueki	2789
Cancer Genet Cytogenet	2362
Leuk Lymph	2226
Bone Marrow Transplant	2183

**Table 9:** 

SELECT	'Berkeley' as institution, count(pmid) as num_of_publications
FROM	medline_citation
WHERE	CONTAINS(article_affiliation,'"Berkeley"') = 1
AND	date_created > current date – 3 years
UNION	
SELECT	'Stanford' as institution, count(pmid) as num_of_publications
FROM	medline_citation
WHERE	CONTAINS(article_affiliation,'"Stanford"') = 1
AND	date_created > current date – 3 years;

**Table 10:** 

Berkeley	2623
Stanford	4226
